# Clinical significance of cefazolin inoculum effect in serious MSSA infections: a systematic review

**DOI:** 10.1093/jacamr/dlae069

**Published:** 2024-05-06

**Authors:** Calvin Ka-Fung Lo, Ashwin Sritharan, Jiesi Zhang, Nicole Li, Cindy Zhang, Frank Wang, Mark Loeb, Anthony D Bai

**Affiliations:** Department of Pathology and Laboratory Medicine, University of British Columbia, Vancouver, BC, Canada; Michael G. DeGroote Undergraduate School of Medicine, McMaster University, Hamilton, ON, Canada; Faculty of Health Sciences, Queen’s University, Kingston, ON, Canada; Faculty of Arts and Sciences, Queen's University, Kingston, ON, Canada; Faculty of Health Sciences, McMaster University, Hamilton, ON, Canada; Michael G. DeGroote Undergraduate School of Medicine, McMaster University, Hamilton, ON, Canada; Department of Pathology and Molecular Medicine, McMaster University, Hamilton, ON, Canada; Division of Infectious Diseases, Department of Medicine, Queen’s University, Kingston, ON, Canada

## Abstract

**Background:**

The cefazolin inoculum effect (CzIE) is a phenomenon whereby some MSSA isolates demonstrate resistance to cefazolin when a high bacterial inoculum is used for susceptibility testing. The clinical significance of this phenotypic phenomenon remains unclear. We conducted a systematic review to answer the following question: In patients with serious MSSA infection treated with cefazolin, does infection due to CzIE-positive MSSA isolates result in worse clinical outcomes than infection due to CzIE-negative MSSA isolates?

**Methods:**

Ovid MEDLINE, Embase, Cochrane CENTRAL, medRxiv and bioRxiv were searched from inception until 12 April 2023. Studies were included if they tested for CzIE in clinical isolates from MSSA infections in humans. Two independent reviewers extracted data and conducted risk-of-bias assessment. Main outcomes were treatment failure and mortality. Pooling of study estimates was not performed given the heterogeneity of patient populations and outcome definitions.

**Results:**

Twenty-three observational studies were included. CzIE presence amidst MSSA isolates ranged from 0% to 55%. There was no statistically significant mortality difference in two studies that compared MSSA infections with and without CzIE, with ORs ranging from 0.72 to 19.78. Of four studies comparing treatment failure, ORs ranged from 0.26 to 13.00. One study showed a significantly higher treatment failure for the CzIE group, but it did not adjust for potential confounders.

**Conclusions:**

The evidence on CzIE is limited by small observational studies. In these studies, CzIE did not predict higher mortality in MSSA infections treated with cefazolin. Our findings do not support CzIE testing in clinical practice currently.

## Background

*Staphylococcus aureus* can cause serious infections, defined as bacteraemia, pneumonia, pleural space infection, endocarditis, CNS infection, native bone or joint infection, prosthetic joint infection and deep abscesses (visceral organ and intramuscular abscesses).^1–3^ Treatment of MSSA infections entails anti-staphylococcal penicillins (e.g. cloxacillin, nafcillin) or a first-generation cephalosporin such as cefazolin.^4^ Although advantages of cefazolin use include more convenient dosing frequency and a reduced risk of nephrotoxicity compared with anti-staphylococcal penicillins such as cloxacillin, concerns exist regarding the presence of a cefazolin inoculum effect (CzIE).^5^

When CzIE is present, it suggests the possibility of increased resistance against cefazolin when there is presence of higher bacterial burden. CzIE is an *in vitro* phenomenon whereby an MSSA isolate is identified as being susceptible to cefazolin when a standard bacterial concentration is tested, but resistant to cefazolin (i.e. elevation in MIC against cefazolin) when a higher bacterial concentration is used for antibiotic susceptibility testing. More specifically, CzIE is defined as a MIC ≤8 mg/L at standard bacterial inoculum of 5 × 10^5^ cfu/mL, and ≥16 mg/L at a higher bacterial inoculum of 5 × 10^7^ cfu/mL (i.e. the effect is proportionate to the bacterial inoculum present at site of infection).^6^ The CzIE phenomenon is potentially mediated by the β-lactamase enzyme (*blaZ)* gene encoding type A and C β-lactamases in MSSA isolates.^7^ Reportedly, up to 25% of MSSA isolates exhibit CzIE, based on a North American study of 305 blood culture isolates.^6^ The reference standard for detecting CzIE is broth microdilution, which requires technical expertise beyond that available in routine microbiology laboratories.^8^ In routine practice, cefazolin susceptibility is inferred from oxacillin or cefoxitin susceptibility testing, molecular detection of *mecA* or *mecC*, or PBP2a assay as per CLSI guidelines.^8^ Thus, routine cefazolin susceptibility testing would not detect CzIE.^8^

For CzIE-positive deep-seated MSSA infections (endocarditis, bone and joint infection, deep-seated abscesses, osteomyelitis or pneumonia)^9^ with a high bacterial burden being treated with cefazolin, there is a theoretical concern that cefazolin will be hydrolysed due to increased production of *blaZ* β-lactamases leading to treatment failure.^10^ However, the exact mechanism of CzIE and connection with clinical outcomes when detected remains unclear at present.

To our knowledge, no systematic review has evaluated whether the CzIE is a risk factor for poor patient outcomes with cefazolin treatment. We conducted a systematic review to answer the following question: In patients with serious MSSA infections (bacteraemia, pneumonia, pleural space infection, CNS infection, endocarditis, native bone or joint infection, prosthetic joint infection or deep abscesses) who were treated with cefazolin, does infection due to MSSA isolates that show CzIE result in worse clinical outcomes than infection due to MSSA isolates without CzIE?

Secondary objectives were to describe: (i) the proportion of MSSA isolates that displayed CzIE across studies; (ii) the diagnostic testing properties of predictors for CzIE; and (iii) the comparison of outcomes in MSSA serious infections with CzIE treated with cefazolin versus an anti-staphylococcal penicillin.

## Methods

### Protocol and registration

This systematic review was prospectively registered on the International Prospective Register of Systematic Reviews (PROSPERO) database (CRD42023420251).

### Search strategy

Studies were identified by searches across five databases: Ovid MEDLINE, Embase, Cochrane CENTRAL, medRxiv and bioRxiv. The search date range was from inception until 12 April 2023. In collaboration with a research librarian at the University of British Columbia, we developed a search strategy using relevant MeSH search terms to optimize search results (Figure S1, available as Supplementary data at *JAC-AMR* Online).

### Eligibility criteria and exclusion criteria

We included all studies published in any language including observational studies (cohort studies, case-control studies, cross-sectional studies) and randomized controlled trials (RCTs) conducted on humans with MSSA infections or studies done on MSSA clinical isolates for which CzIE testing was done. Although this may capture non-serious MSSA infections, this strategy ensured we did not miss studies that cover our secondary objectives.

Systematic reviews, meta-analyses, case reports, commentaries, letters, abstracts, conference reports or reports of only study design were excluded. Studies that focused strictly on animal models were excluded. Studies primarily focusing on MRSA were excluded as well. If multiple published studies were based on the same patient group and reported the same mortality or treatment failure outcome, only the study describing the largest patient group was selected so that the same patient would not be double counted in the systematic review.

### Data extraction

Abstracts were screened by two blinded independent authors to identify potentially relevant studies for full-text screening and review. Subsequently, two blinded independent authors read and reviewed the full text for data extraction. Disagreements during study selection and data extraction process were resolved by a third reviewer.

Data were collected on the following variables:

- Journal article information: author, publication year
- Study information: study type, study location, sample size
- Patient information: demographics, risk factors
- Infection: source of clinical isolate, infectious syndrome
- CzIE testing result and predictors for CzIE used: we defined CzIE as MIC ≤8 mg/L at standard inoculum (∼5 × 10^5^ cfu/mL) and MIC ≥16 mg/L at high inoculum (∼5 × 10^7^ cfu/mL).^6^ Any surrogate markers for predicting cefazolin resistance or elevated MIC were noted (outside of reference standard of broth microdilution).
- Treatment: antibiotic used as definitive treatment for MSSA infection

### Outcomes

The co-primary outcomes were mortality and treatment failure, however defined by the study. Mortality could be all-cause or attributable within the time frame as reported in the study. Treatment failure could include death, complication related to infection, discontinuation of antibiotic treatment due to adverse effects, switch to another antibiotic due to lack of clinical response, and/or recurrence of infection.

### Risk-of-bias assessment

Two reviewers independently assessed the risk of bias with the plan of using the tool that would be most appropriate for the study design and research question: Newcastle-Ottawa Scale (NOS)^11^ for observational studies, Cochrane Risk-of-Bias (RoB) Version 2^12^ for RCTs, Joanna Briggs Institute (JBI) prevalence studies checklist^13^ for lab-testing studies, and QUADAS-2 tool^14^ for diagnostic accuracy studies.

### Statistical analysis

The proportion of CzIE in samples was calculated for each study. In a *post hoc* analysis, we described the prevalence of CzIE in studies categorized by country and continent.

For diagnostic parameters of CzIE predictors, we calculated the sensitivity, specificity and likelihood ratios with corresponding 95% CIs for each study in comparison with the listed reference standard (i.e. broth micro or macro-dilution). In 2 × 2 tables with a zero in one cell, 0.5 was added to all cells before doing the calculations.^15^

There was significant clinical heterogeneity in terms of how the patient population and outcomes were defined across studies. Therefore, only a descriptive analysis of individual studies was done. For each study, we compared the mortality and treatment failure as defined by the study between MSSA isolates with CzIE and MSSA isolates without CzIE in patients with MSSA infections who were treated with cefazolin. We calculated the unadjusted OR with MSSA isolates without CzIE as the reference and 95% CI for mortality and treatment failure. We also reported the adjusted ORs if provided by the study.

Data analysis was performed using the statistical software SPSS ver27 2020 and R.

## Results

### Study selection and description of included studies

**Figure 1.**
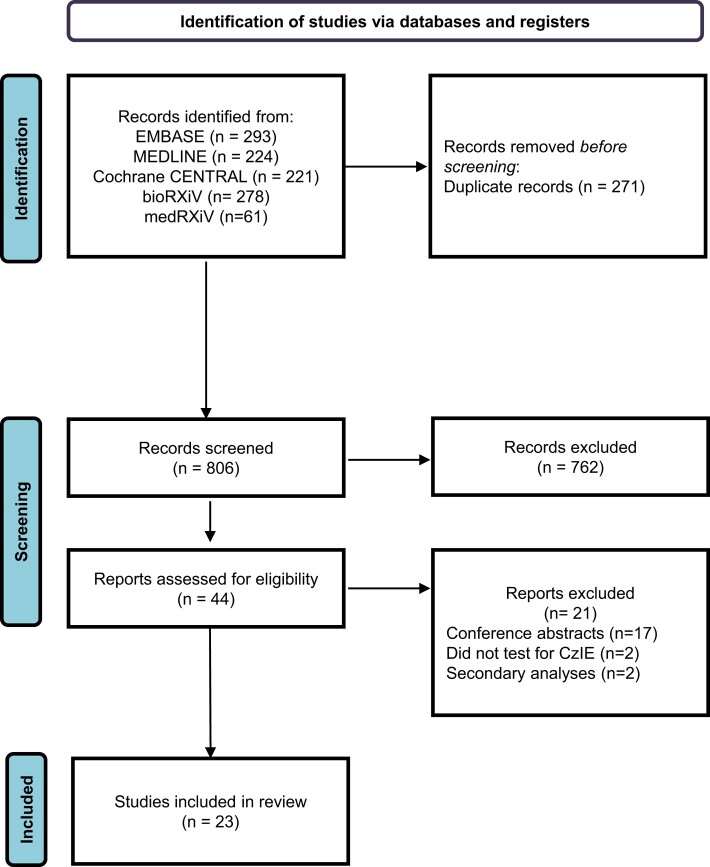
Flow diagram for inclusion of studies.

The literature search yielded 1077 records (Figure 1). A total of 271 duplicates were removed, resulting in 806 unique records that were screened. After abstract screening and full-text reading, 23 studies were included in the analysis (Table 1).^6–8,10,16–34^ Excluded studies and corresponding exclusion reasons are listed in Table S1.

**Table 1.** Study characteristics: study type, geographical location, cefazolin inoculum effect (CzIE) proportion and infection type across included studies (*N* = 23)

Study reference no.	Study design Centre Year Location	Patient population CzIE-positive group CzIE-negative group	Primary outcome	Conclusion
16	Retrospective cohort Single centre 2013–2018 France	Patients with MSSA endocarditis (*N* = 51) CzIE-positive group (*n* = 4) treated with cefazolin CzIE-negative group (*n* = 16) treated with cefazolin	Persistent bacteraemia on treatment ≥72 h Clinical failure at 3 mo based on worsening on therapy, septic embolism, discontinuation of treatment due to adverse events, relapse or death	CzIE was not associated with increased risk for clinical failure on treatment
17	Lab testing Multicentre 2010–2014, 2018–2019 Latin America	Patients with MSSA bacteraemia (*N* = 690) CzIE-positive group (*n* = 278) CzIE-negative group (*n* = 412)	Association between allotypes of BlaZ and CzIE	Certain allotypes were more associated with CzIE, whereas others were not
18	Retrospective cohort Multicentre 2011–2018 USA	Patients with haematogenous MSSA osteomyelitis (*N* = 250) CzIE-positive group (*n* = 36) CzIE-negative group (*n* = 214)	Progression to chronic osteomyelitis	CzIE is independently associated with progression to chronic osteomyelitis irrespective of final antibiotic choice
19	Retrospective cohort Single centre 2008–2011 South Korea	Patients with MSSA bacteraemia (*N* = 220), of which 77 patients were treated with cefazolin CzIE-positive group (*n* = 29) CzIE-negative group (*n*= 191)	Treatment failure defined as: (i) a change of antibiotics due to clinical failure during treatment, (ii) relapse of MSSA infection after apparently successful completion of treatment or (iii) MSSA bacteraemia-attributable mortality	CzIE was not associated with increased risk for clinical failure on treatment
6	Lab testing Multicentre 2019 USA, Canada	Patients with MSSA bacteraemia (*N* = 305) CzIE-positive group (*n* = 57) CzIE-negative group (*n* = 248)	Prevalence of CzIE and its association with β-lactamase types	CzIE was present in up to 25% of clinical MSSA isolates. Most common *blaZ* β-lactamases found in CzIE strains were type A and C
20	Lab testing Single centre 1976 USA	MSSA clinical isolates not otherwise specified (*N* = 100)	Association between inoculum size and MIC of different cephalosporins	Different cephalosporins are inactivated with increasing bacterial inoculum resulting in high MICs. The clinical significance of this phenomenon is not known
21	Lab testing Multicentre 2004–2013 South Korea	MSSA blood isolates (*N* = 113) Of 17 strains positive for type A *blaZ* gene, 10 tested positive for CzIE and 7 tested negative	Association between type A *blaZ* polymorphism and CzIE	The SNP at codon 226 and 229 encoded by *blaZ* gene is closely associated with CzIE
7	Retrospective cohort Multicentre 2004–2013 South Korea	Patients with MSSA bacteraemia treated with cefazolin (*N* = 113) Of 113 cases, 23 (20.4%) displayed CzIE based on increase in MIC to ≥16 mg/L at high inoculum	Treatment failure defined as: (i) switching of antibiotics due to the clinician’s decision of the treatment failure; (ii) recurrence of MSSA infection; or (iii) MSSA bacteraemia-associated mortality	CzIE may be associated with persistent bacteraemia, but not significantly associated with treatment failure
22	Prospective cohort Multicentre 2013–2015 South Korea	Patients with MSSA bacteraemia (*N* = 242) In the propensity score-matched cohort of 110 cases, 24 were CzIE positive and 86 were CzIE negative	Treatment failure defined as: (i) switching antibiotics because treatment had failed in the clinician's opinion; (ii) premature discontinuation of antibiotics because of adverse effects; (iii) all-cause mortality within 1 mo; or (iv) recurrence or relapse of MSSA infection within 3 mo of treatment completion	CzIE is associated with cefazolin treatment failure for MSSA bacteraemia
23	Lab testing Single centre 2014–2017 South Korea	MSSA blood isolates (*N* = 195) CzIE-positive group (*n* = 23) CzIE-negative group (*n* = 172)	Association between *blaZ* genotype and CzIE	Type A *blaZ* genotype with *agr* type III could be a useful indicator to genetically differentiate CzIE-positive MSSA isolates
24	Retrospective cohort Multicentre 2010–2010 USA	MSSA isolates from bacteraemia (*N* = 185), of which 8 (4%) demonstrated CzIE based on increase in MIC to ≥16 mg/L at high inoculum	Prevalence of *blaZ* gene types and CzIE	*blaZ* gene was present in 142/185 (77%) isolates. There were 50 isolates that had ≥4-fold increase in MIC and 8 (4%) had a non-susceptible cefazolin MIC at high inoculum, which were all type A *blaZ* strains
25	Prospective cohort Multicentre 2011–2014 Argentina	Patients with MSSA bacteraemia (*N* = 77) CzIE-positive group (*n* = 42) CzIE-negative group (*n* = 35)	30 d mortality, treatment failure	CzIE was associated with increased 30 d mortality when cephalosporins are used as first-line therapy for MSSA bacteraemia
26	Lab testing Multicentre 2011 Japan	MSSA clinical isolates from sputum, pharynx and blood (*N* = 49)	Association between inoculum and MIC for different antibiotics	There were small fluctuations in cefazolin MIC when increasing inoculum was used
27	Lab testing Multicentre 1981–2017 Canada	MSSA isolates from sputum culture in non-cystic fibrosis bronchiectasis (*N* = 60) Of 60 isolates, there were no cases of pronounced CzIE based on increase in MIC to ≥16 mg/L at high inoculum	To describe the prevalence of inoculum effect for different antibiotics	Inoculum-related resistance was relevant for commonly used antibiotics such as cefazolin and piperacillin/tazobactam
10	Prospective cohort Multicentre Not specified Not specified	Clinical isolates from endocarditis (*n* = 29), pneumonia (*n* = 29), skin and soft tissue infection (*n* = 28), and bacteraemia (*n* = 12). Of the 98 isolates, 19 (19.2%) displayed CzIE	Association of CzIE with the type of β-lactamases Treatment failure	CzIE was found in 19 cases, of which 10 were type C β-lactamase producers and 9 were type A β-lactamase producers. There was an association between CzIE and cefazolin failure in haemodialysis patients with bacteraemia
28	Diagnostic accuracy Multicentre 2011–2019 Latin America and USA	MSSA isolates from blood culture (*N* = 689) CzIE-positive group (*n* = 257) CzIE-negative group (*n* = 432)	Diagnostic accuracy of a colorimetric test to detect CzIE	Rapid colorimetric test can accurately detect CzIE with sensitivity of 82.5% and specificity of 88.9%
8	Lab testing Multicentre 2001–2008 South America	MSSA isolates from bloodstream (*n* = 296) and osteomyelitis (*n* = 68) CzIE-positive group (*n* = 131) CzIE-negative group (*n* = 233)	Prevalence of CzIE across South American hospitals	There is a high prevalence of CzIE associated with type A β-lactamase in Colombia and Ecuador
29	Lab testing Single centre 1975 USA	Clinical MSSA isolates (*N* = 118) not otherwise specified. CzIE status was not specified	To determine the inoculum effect on the anti-staphylococcal β-lactams	Of tested β-lactams, cefazolin was more susceptible to inoculum effect than other cephalosporins
30	Lab testing Single centre 2012–2014 Japan	MSSA isolates from blood culture (*N* = 52), of which 3 (5.8%) had pronounced CzIE	Inoculum effect to different β-lactams	Inoculum effect was found for cefazolin and ampicillin/sulbactam, but not cefotaxime, ceftriaxone, imipenem and meropenem
31	Prospective cohort Multicentre 2013–2015 South Korea	Patients with MSSA bacteraemia (*N* = 303) CzIE-positive group (*n* = 61) CzIE-negative group (*n* = 242)	Characteristics of CzIE-positive isolates	Erythromycin and clindamycin resistance were predictors of CzIE
32	Lab testing Single centre 2019 Russia	MSSA isolates from skin/soft tissue infection (*N* = 80), of which 2 (2.5%) displayed CzIE based on increase in MIC to ≥16 mg/L at high inoculum	Prevalence of CzIE and its association with penicillin resistance	CzIE is associated with penicillin resistance and β-lactamase *blaZ* gene
33	Lab testing Multicentre 2014–2015 USA	MSSA clinical isolates from any sites (*N* = 269), of which 5 (3%) displayed pronounced CzIE	Association with *blaZ* gene types and CzIE in MSSA	The local prevalence of pronounced CzIE was low
34	Prospective cohort Multicentre 2011–2012 South Korea	Patients with MSSA bacteraemia (*N* = 146) CzIE-positive group (*n* = 16) CzIE-negative group (*n* = 130)	Association of CzIE with β-lactamase types and dysfunctional accessory gene regulator (*agr*)	CzIE was associated with type A β-lactamase and dysfunctional *agr*

### Risk-of-bias assessment

Risk-of-bias assessment is described in Table S2 for 10 cohort studies, Table S3 for 12 lab-testing studies and Table S4 for one diagnostic accuracy study. For cohort studies, each study was rated on an overall score from 1 to 9, with 6–9 being high quality, 3–5 being fair and 0–2 being poor quality.^11^ Nine studies scored at least 6 points. The remaining study had a score of 4. Of note, there were no eligible RCTs and hence the RoB Version 2 tool was not applicable in our assessment.

### Proportion of MSSA isolates that displayed CzIE

There were 19 studies that reported the proportion of MSSA isolates displaying CzIE as defined by MIC ≤8 mg/L at standard inoculum and MIC ≥16 mg/L at high inoculum (Table 2). The median number of isolates per study was 185 (range 51 to 690), with a median of 14.4% of isolates being positive for CzIE (range 0% to 54.5%). The proportion of CzIE in MSSA ranged from 0% to 18.7% in North American countries, 36.0% to 54.5% in South American countries, 2.5% to 11.0% in European countries, and 5.8% to 21.8% in Asian countries.

**Table 2.** Proportion of MSSA isolates that tested positive for cefazolin inoculum effect (CzIE) in studies categorized by country and continent^a^

Continent	Country	Study reference	Isolates tested for CzIE, *N*	Isolates with CzIE, *n* (%)
North America	USA	18	250	36 (14.4)
	USA	24	185	8 (4.3)
	USA	33	269	7 (2.6)
	USA, Canada	6	305	57 (18.7)
	Canada	27	60	0 (0)
South America	Argentina	25	77	42 (54.5)
	Colombia, Ecuador, Peru, Venezuela	8	364	131 (36.0)
North and South America	Mexico, Colombia, Peru, Argentina, Ecuador, Chile, Brazil, Guatemala, Venezuela	17	690	278 (40.3)
	Mexico, Colombia, Peru, Argentina, Ecuador, Chile, Brazil, Guatemala, Venezuela, USA	28	689	257 (37.3)
Europe	France	16	51	2 (3.9)
	France	34	146	16 (11.0)
	Russia	32	80	2 (2.5)
Asia	South Korea	19	220	29 (13.2)
	South Korea	7	113	23 (20.4)
	South Korea	22	110	24 (21.8)
	South Korea	23	195	23 (11.8)
	South Korea	31	303	61 (20.1)
	Japan	30	52	3 (5.8)

^a^Study reference 10 was not included because the country of origin for the MSSA isolates was unclear. In this study, 19/98 (19.2%) displayed CzIE. Study reference 20 was not included because the testing method and definition for CzIE are different from our definition. Study reference 21 was not included because it tested only MSSA isolates that tested positive for type A *blaZ* gene.

### Surrogate predictors for CzIE

Three studies reported predictors for CzIE that allowed calculation of diagnostic properties. Surrogate predictors included erythromycin resistance, clindamycin resistance, and a rapid colorimetric testing. Diagnostic properties are summarized in Table 3. The rapid colorimetric test specifically designed for detection of CzIE^28^ had the best combination of sensitivity and specificity based on the point estimate and CI.

**Table 3.** Diagnostic properties of surrogate predictors for cefazolin inoculum effect (CzIE)

Screening test	Study reference	True positive	False negative	False positive	True negative	Sensitivity, % (95% CI) Specificity, % (95% CI) Positive likelihood ratio (95% CI) Negative likelihood ratio (95% CI)
Erythromycin resistance	31	20	41	22	220	Sn: 32.8 (22.3–45.3)
						Sp: 90.9 (86.6–93.9)
						PLR: 3.6 (2.1–6.2)
						NLR: 0.74 (0.62–0.89)
Clindamycin resistance	18	9	27	20	194	Sn: 25.0 (13.8–41.1)
						Sp: 90.7 (86.0–93.9)
						PLR: 2.7 (1.3–5.4)
						NLR: 0.83 (0.68–1.00)
	31	13	48	17	225	Sn: 21.3 (12.9–33.1)
						Sp: 93.0 (89.0–95.6)
						PLR: 3.0 (1.6–5.9)
						NLR: 0.85 (0.74–0.97)
Rapid colorimetric test	28	212	45	48	384	Sn: 82.5 (77.4–86.6)
						Sp: 88.9 (85.6–91.5)
						PLR: 7.4 (5.7–9.8)
						NLR: 0.20 (0.15–0.26)

NLR, negative likelihood ratio; PLR, positive likelihood ratio; Sn, sensitivity; Sp, specificity.

### Mortality and treatment failure

Only two studies reported mortality outcomes (Table 4). The mortality definition varied in terms of timepoint, which ranged from 1 month to 3 months. The wide 95% CIs reflect small number sizes and inconclusive results. No study showed a significant difference in mortality between CzIE-positive and -negative MSSA infections.

**Table 4.** Clinical outcomes comparing CzIE-positive versus CzIE-negative isolates for MSSA infections treated with cefazolin

Outcome	Study	Definition	Events in CzIE-positive group	Events in CzIE-negative group	OR for CzIE (95% CI)
Mortality	19	30 d all-cause mortality	1/10 (10.0%)	9/67 (13.4%)	OR: 0.72 (0.08–6.35)
	19	12 wk all-cause mortality	2/10 (20.0%)	12/67 (17.9%)	OR: 1.15 (0.22–6.09)
	22	1 mo all-cause mortality	2/13 (15.4%)	0/45 (0%)	OR: 19.78 (0.89–441.14)
	22	3 mo all-cause mortality	2/13 (15.4%)	0/45 (0%)	OR: 19.78 (0.89–441.14)
Treatment failure	19	Antibiotic switch, relapse post-treatment or infection related mortality	0/10 (0%)	10/67 (14.9%)	OR: 0.26 (0.01–4.80)
	7	Antibiotic switch, infection recurrence or infection-related death within 12 wk	Not reported	Not reported	OR: 1.39 (0.45–4.32) aOR: 1.30 (0.35–4.88)
	22	Antibiotic switch, death within 1 mo, recurrence within 3 mo, discontinuation from adverse events	8/13 (61.5%)	13/45 (28.9%)	OR: 3.93 (1.08–14.31)
	10	Relapse and infection-related mortality (no time period was specified for outcome)	3/3 (100%)	3/9 (33.3%)	OR: 13.00 (0.51–330.50)

aOR, adjusted odds ratio, otherwise OR refers to unadjusted odds ratios; CzIE, cefazolin inoculum effect.

Four studies reported treatment failure outcomes (Table 4). The definition for treatment failure varied across studies. A single study found a statistically significant increase in treatment failure for CzIE-positive isolates with an OR of 3.93 (95% CI: 1.08 to 14.31) (Table 4).^22^ In this study, there was no adjustment for potential confounders in the subgroup analysis when the CzIE-positive group was compared with the CzIE-negative group.^22^

### Cefazolin versus anti-staphylococcal penicillin for CzIE-positive MSSA infections

One study compared the mortality and treatment failure between an anti-staphylococcal penicillin and cefazolin for CzIE-positive MSSA infections.^22^ At the end of 1 month and 3 months, 2/13 (15.4%) patients in the cefazolin group and 1/11 (9.1%) patients in the nafcillin group died (OR 1.82; 95% CI: 0.14 to 23.26).^22^ Treatment failure, defined as discontinuation of antibiotics due to adverse effects, antibiotic change due to clinical failure, death within 1 month and recurrence, occurred in 8/13 (61.5%) for the cefazolin group and 4/11 (36.4%) patients for the nafcillin group (OR 2.80; 95% CI: 0.53 to 14.74).

## Discussion

Our results show that the clinical impact of CzIE is based on small observational studies that provide poor quality evidence. Overall, the proportion of MSSA infections displaying CzIE ranged from 0% to 55% across studies. No study found a significant difference in mortality between CzIE-positive and -negative MSSA infections treated with cefazolin. All but one study found no significant difference in treatment failure between CzIE-positive and -negative MSSA infections treated with cefazolin. Therefore, there is a lack of evidence to support that CzIE is clinically important currently.

Strengths of our review included a comprehensive search of multiple databases that included preprints and had no language restrictions. There was also rigorous screening and data collection by two independent reviewers for each study.

Limitations included the very limited sample size pool of four studies that reported mortality and treatment outcomes.^7,10,19,22^ Most studies did not adjust for potential confounders. There was significant heterogeneity across studies for types of infections and definition of outcomes. Only one study reported clinical outcomes for anti-staphylococcal penicillins.^22^ It is important to compare the effectiveness of cefazolin versus anti-staphylococcal penicillin in the treatment of MSSA infection with CzIE. The rationale is that if MSSA infections with CzIE treated with cefazolin had worse outcomes than MSSA infections without CzIE treated with cefazolin, there may be a reason other than cefazolin treatment and CzIE for the difference in outcome. MSSA isolates that display CzIE may also have other intrinsic bacterial characteristics that make the isolates more virulent and the infection more deadly regardless of antibiotic treatment choice. If that is the case, then MSSA with CzIE treated with cefazolin versus an anti-staphylococcal penicillin would have similar outcomes.

Large studies are needed to provide higher quality evidence on whether CzIE is clinically important. The ideal study should include serious and deep-seated infections in which CzIE may be clinically relevant. MSSA infections with CzIE treated with cefazolin should be compared with MSSA infections without CzIE treated with cefazolin as well as MSSA infections with CzIE treated with an anti-staphylococcal penicillin. Lastly, adjustment should be made for potential confounders.

### Conclusions

In conclusion, there is very low quality of evidence at present that does not support the theory that CzIE translates to worse outcomes in terms of mortality or treatment failure for serious MSSA infections being treated with cefazolin. Thus, our study supports the CLSI recommendation that CzIE should not be tested in clinical settings outside of research until there is more evidence to suggest otherwise.^35,36^ Clinical microbiology laboratories should avoid routine testing for CzIE when pursuing microbiological workup of MSSA clinical isolates, because the current evidence does not support the use of CzIE results when making clinical treatment decisions for MSSA infections.

## Supplementary Material

dlae069_Supplementary_Data

## Data Availability

Data and material are available and will be provided upon request from the corresponding author.

## Supplementary data

Figure S1 and Tables S1 to S4 are available as Supplementary data at *JAC-AMR* Online.
