# Supplementary material for: Clinical significance of cefazolin inoculum effect in serious MSSA infections: a systematic review
Source: JAC Antimicrob Resist. 2024 May 6;6(3):dlae069. doi: 10.1093/jacamr/dlae069 (PMC11073751; doi:10.1093/jacamr/dlae069)
Supplement: dlae069_Supplementary_Data [file dlae069_supplementary_data.docx]

**Figure S1: Search Strategy**

1) MeSH terms and keywords: *Staphylococcus aureus*, Staphylococcal infections, Methicillin-S, MSSA, Bacteremia

2) MeSH terms and keywords: Cefazolin, cephalosporins,

3) MeSH terms of keywords: CzIE, Inoculum

Example of search strategy for EMBASE detailed as below:

1 Staphylococcus aureus.mp.

2 Staphylococcus aureus/

3 S* aureus.mp.

4 Staphylococcal Infections/

5 Methicillin/

6 Methicillin-S*.mp.

7 Bacteremia/

8 bacteremia.mp.

9 MSSA.mp.

10 or/1-9

11 cefazolin.mp.

12 Cefazolin/

13 cephalosporin*.mp.

14 Cephalosporins/

15 or/11-14

16 CzIE.mp.

17 inoculum.mp.

18 16 or 17

19 10 and 15 and 18

20 Cefazolin inoculum effect.mp.

21 19 or 20

**Table S1. Excluded studies and rationale for exclusion (n=21 studies).**

| Ref | Reason for Exclusion |
| --- | --- |
| Carvajal 2019 | Conference Abstract |
| Carvajal 2018 | Conference Abstract |
| Diaz 2019 | Conference Abstract |
| Du 2022 | Conference Abstract |
| Erdman 2022 | Conference Abstract |
| Gomez-Villegas 2020 | Conference Abstract |
| Hess 2023 | Did not include cefazolin inoculum effect testing |
| Laurent 2018 | Conference Abstract |
| Lee 2011 | Did not include cefazolin inoculum effect testing |
| McNeil 2019 | Conference Abstract |
| McNeil 2020 | Duplicate/secondary analysis of McNeil 2020^18^ |
| Mersinger 2020 | Conference Abstract |
| Pinargote-Cornejo 2020 | Conference Abstract |
| Quarshie 2016 | Conference Abstract |
| Smelter 2020 | Conference Abstract |
| Svishchuk 2022 | Conference Abstract |
| Svishchuk 2022 | Conference Abstract |
| Uloa 2019 | Conference Abstract |
| Villegas 2022 | Conference Abstract |
| Wang 2016 | Conference Abstract |
| Wi 2015 | Duplicate/Secondary Analysis of Wi 2015^34^ |

**Table S2. Risk of Bias assessment based on Newcastle-Ottawa Scale for cohort studies**

| Ref # | Selection: Cohort representativeness | Selection: non-exposed cohort | Ascertainment exposure | Outcome absent at start | Cohort comparability control - (most important factor) | Cohorts Comparability control- (additional factor) | Assessment | Outcome - Adequate follow-up length | Outcome- adequate cohort follow-up | Total Score |
| --- | --- | --- | --- | --- | --- | --- | --- | --- | --- | --- |
| 16 | 0 (selected use group) | 1 (same as exposed) | 1 (secure record) | 1 (Y) | 0 (N) | 0 (N) | 1 (record linkage) | 1 (Y) | 1 (complete follow-up) | 6 |
| 18 | 1 (somewhat) | 1 (same as exposed) | 1 (secure record) | 1 (Y) | 1 (Y) | 1 (Y) | 0 (no descript) | 1 (Y) | 0 (follow-up inadequate, no descript for lost) | 7 |
| 19 | 1 (truly rep) | 1 (same as exposed) | 1 (secure record) | 1 (Y) | 0 (N) | 0 (N) | 1 (record linkage) | 1 (Y) | 0 (follow-up inadequate, no descript for lost) | 6 |
| 7 | 1 (somewhat) | 1 (same as exposed) | 1 (secure record) | 1 (Y) | 1 (Y) | 1 (Y) | 1 (record linkage) | 1 (Y) | 0 (no statement) | 8 |
| 22 | 1 (truly rep) | 1 (same as exposed) | 1 (secure record) | 1 (Y) | 1 (Y) | 1 (Y) | 1 (record linkage) | 1 (Y) | 0 (no statement) | 8 |
| 24 | 1 (truly rep) | 1 (same as exposed) | 1 (secure record) | 1 (Y) | 0 (N) | 0 (N) | 1 (record linkage) | 1 (Y) | 0 (follow-up inadequate, no descript for lost) | 6 |
| 25 | 1 (truly rep) | 1 (same as exposed) | 1 (secure record) | 1 (Y) | 1 (Y) | 1 (Y) | 1 (record linkage) | 1 (Y) | 1 (complete follow-up) | 9 |
| 10 | 1 (somewhat) | 1 (same as exposed) | 1 (secure record) | 0 (N) | 0 (N) | 0 (N) | 1 (independent blind assessment) | 0 (N) | 0 (no statement) | 4 |
| 31 | 1 (somewhat) | 1 (same as exposed) | 1 (secure record) | 1 (Y) | 1 (Y) | 1 (Y) | 1 (record linkage) | 1 (Y) | 1 (unlikely bias for lost f/u) | 9 |
| 34 | 1 (truly rep) | 1 (same as exposed) | 1 (secure record) | 1 (Y) | 1 (Y) | 0 (N) | 1 (record linkage) | 1 (Y) | 1 (complete follow-up) | 8 |

**Table S3. Risk of bias assessment based on JBI prevalence tool for laboratory testing studies**

| Study | Sample frame | Sampling | Sample size | Description of subjects and setting | Sufficient coverage | Valid method to identify condition | Reliable and standard way to measure condition | Appropriate statistical analysis | Response rate adequate |
| --- | --- | --- | --- | --- | --- | --- | --- | --- | --- |
| 6 | Unclear | No | Yes | No | Yes | Yes | Yes | Yes | N/A |
| 8 | Unclear | No | Yes | No | Yes | Yes | Yes | Yes | N/A |
| 20 | Unclear | No | No | No | Unclear | No | Yes | No | N/A |
| 17 | Unclear | No | Yes | No | Yes | Yes | Yes | Yes | N/A |
| 21 | Unclear | No | No | No | No | Yes | Yes | Unclear | No |
| 23 | Yes | No | Yes | Yes | Unclear | Yes | Yes | Yes | N/A |
| 26 | Unclear | No | No | No | Unclear | No | No | No | No |
| 27 | Yes | No | No | Yes | Yes | Yes | Yes | Yes | N/A |
| 29 | Unclear | No | Yes | No | Unclear | No | Yes | Yes | N/A |
| 30 | Unclear | No | No | No | Unclear | Yes | Yes | Yes | N/A |
| 32 | Unclear | No | No | No | Unclear | Yes | Yes | Yes | N/A |
| 33 | Unclear | No | Yes | No | No | Yes | Yes | Yes | N/A |

**Table S4. Risk of bias assessment based on QUADAS-2 tool for diagnostic accuracy studies**

| Study | Domain 1: Patient selection | Domain 2: Index test | Domain 3: Reference standard | Domain 4: Flow and timing |
| --- | --- | --- | --- | --- |
| 28 | Risk of bias: Unclear, likely low risk  Consecutive or random sample: Unclear  Case control design avoided: Yes  Inappropriate exclusions avoided: Yes  Applicability: Low concern | Risk of bias: Unclear  Index test interpreted without knowledge of reference standard: Unclear  Pre-specified threshold: Unclear  Applicability: Low concern | Risk of bias: Unclear, likely low risk  Reference standard likely to classify correctly: Yes  Reference standard interpreted without knowledge of index test: Unclear  Applicability: Low concern | Risk of bias: Low risk  Appropriate interval: Yes  All patients receive reference standard: Yes  Patients receive same reference standard: Yes  All patients included in analysis: Yes |
